# Case Report: A novel 3D-printed dual-matrix system for guided fabrication of indirect anterior composite veneers

**DOI:** 10.3389/fdmed.2026.1879301

**Published:** 2026-06-29

**Authors:** Federico Wirz, Maximiliano Cáceres, Isidora Arnés, Gerardo Durán Ojeda

**Affiliations:** 1 Private Practitioner, Buenos Aires, Argentina; 2 Job's Dental Laboratory, Buenos Aires, Argentina; 3 Faculty of Dentistry, Universidad de los Andes, Santiago, Chile

**Keywords:** 3D-Printing, case report, composite resin, dental, laminate veneers, veneers

## Abstract

Resin composite veneers are widely used for esthetic rehabilitation of anterior teeth, offering a minimally invasive approach to restore tooth form and harmony. In indirect techniques, a digital diagnostic wax-up can define the final morphology; however, its accurate transfer to the clinical setting remains challenging and operator-dependent, potentially compromising restoration adaptation and introducing variability at the adhesive interface. This study describes a novel 3D-printed dual matrix system designed to improve the accuracy and reproducibility of the digital wax-up transfer. In the presented case, a 19-year-old patient with anterior diastemas and enamel defects was treated using a fully digital workflow. A diagnostic wax-up was used to fabricate a 3D-printed dual-matrix system with interlocking palatal and vestibular components, enabling guided positioning and controlled seating during the fabrication of the indirect resin composite veneers. The restorations were initially light-cured intraorally for stabilization, then removed and post-cured extraorally, followed by adhesive luting under rubber dam isolation. The proposed system enables a controlled and reproducible transfer of the digital diagnostic wax-up, facilitating the fabrication of indirect resin composite veneers. By improving restoration positioning and seating, it may enhance control of the adhesive interface and the predictability of bonding procedures for indirect resin composite veneers.

## 1 Introduction

Direct anterior restorations with resin composite represent a minimally invasive and esthetic treatment option; however, their clinical success remains highly dependent on operator skill (1, 2). Achieving ideal morphology, seamless optical integration, and appropriate surface characteristics requires precise control of stratification, shade matching, and finishing and polishing procedures, making these restorations inherently technique-sensitive (3, 4).

To improve control over finishing and polishing, and to enhance the mechanical properties of resin composite veneers, techniques involving extraoral refinement have been introduced (5, 6). In these approaches, restorations may be fabricated on models or directly on the teeth without adhesive procedures, allowing their subsequent removal for extraoral refinement, including finishing, polishing, and post-polymerization (7). This additional polymerization increases the degree of conversion, thereby improving both mechanical properties and surface quality, prior to definitive adhesive cementation (8, 9).

From an adhesive standpoint, the quality of the bonded interface is not only dependent on material selection and surface treatment protocols, but also on the accuracy of restoration seating and adaptation. Inadequate positioning or discrepancies during seating may lead to variations in resin cement thickness, incomplete adaptation, and increased polymerization shrinkage stress, ultimately affecting the bonding durability and clinical longevity (10, 11).

Despite these advantages, the technique is typically performed freehand, making the final outcome highly operator-dependent (7). However, conventional freehand approaches and matrix systems may limit precise morphological control and compromise reproducibility, preventing consistent and anatomically accurate outcomes (7, 12, 13). More recently, digitally guided injection technique using transparent matrices and digital workflows have been introduced to improve the transfer accuracy of the diagnostic wax-up and enhance morphological predictability during direct composite restorations (14, 15). Although these approaches have demonstrated promising clinical outcomes, they remain primarily focused on direct restorative procedures.

Moreover, the lack of controlled positioning during restoration placement may introduce variability at the adhesive interface, further compromising the predictability of bonding procedures. Therefore, this article describes a novel 3D-printed dual-matrix system designed to enable controlled, reproducible, and anatomically guided fabrication of anterior indirect composite veneers, integrating a fully digital workflow to enhance clinical predictability.

## 2 Case presentation

The CARE guidelines were followed for the reporting and documentation of this clinical report.

### 2.1 Initial appointment

**Figure 1:**
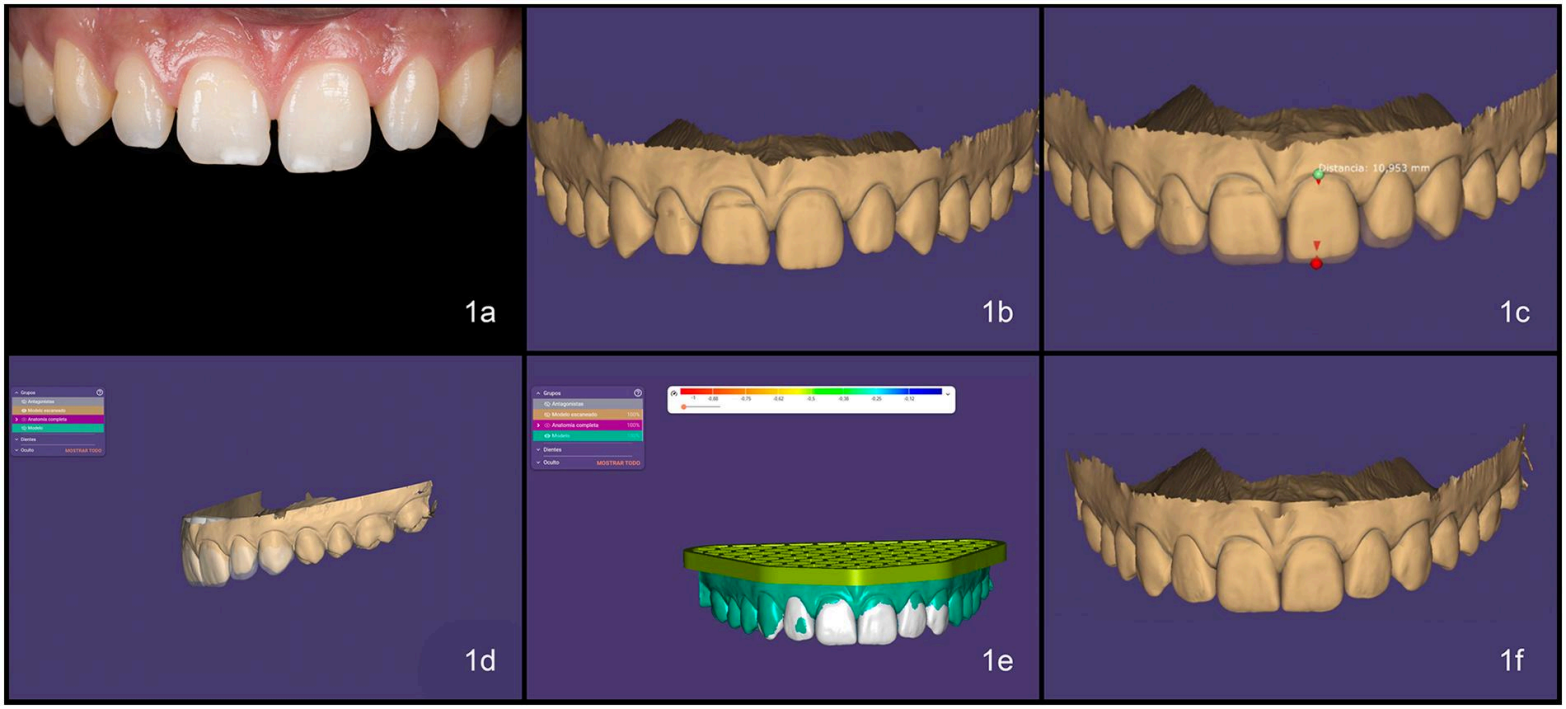
Initial situation and digital design: **(a)** initial condition showing multiple diastemas and enamel defects following orthodontic treatment; **(b)** initial maxillary digital scan; **(c)** digital wax-up superimposition illustrating the selected length of the left maxillary central incisor for reference; **(d)** lateral view of the digital design; **(e)** frontal view highlighting the areas planned for additive restorative material; **(f)** finalized digital treatment planning.

A 19-year-old male patient presented to the private practice of one of the authors with esthetic concerns related to the presence of multiple diastemas between the maxillary incisors. In addition, enamel defects were observed as a consequence of orthodontic fixed appliance removal, particularly affecting teeth #11 and #12 (Figure 1a).

Baseline photographic records were obtained, along with an intraoral scan of the initial condition (Figures 1a,b) using an intraoral scanner (Primescan, DENTSPLY SIRONA). The virtual patient model was exported as an STL file and processed using computer-aided design (CAD) software to develop a digital diagnostic wax-up (Exocad 3.0, GmbH, Darmstadt, Germany).

### 2.2 Diagnostic digital wax-up and 3D-printed dual matrix system fabrication

The length of both maxillary central incisors was initially established (Figure 1c) to define the incisal plane and guide the subsequent design of the lateral incisors and canines. Once the final tooth volumes were determined and the proposed morphology was validated (Figures 1d,e), the digital wax-up was finalized and exported as an STL file for further processing (Figure 1f). The final veneer thickness was determined by the additive digital wax-up and varied according to the amount of space closure and morphological correction required for each tooth.

**Figure 2:**
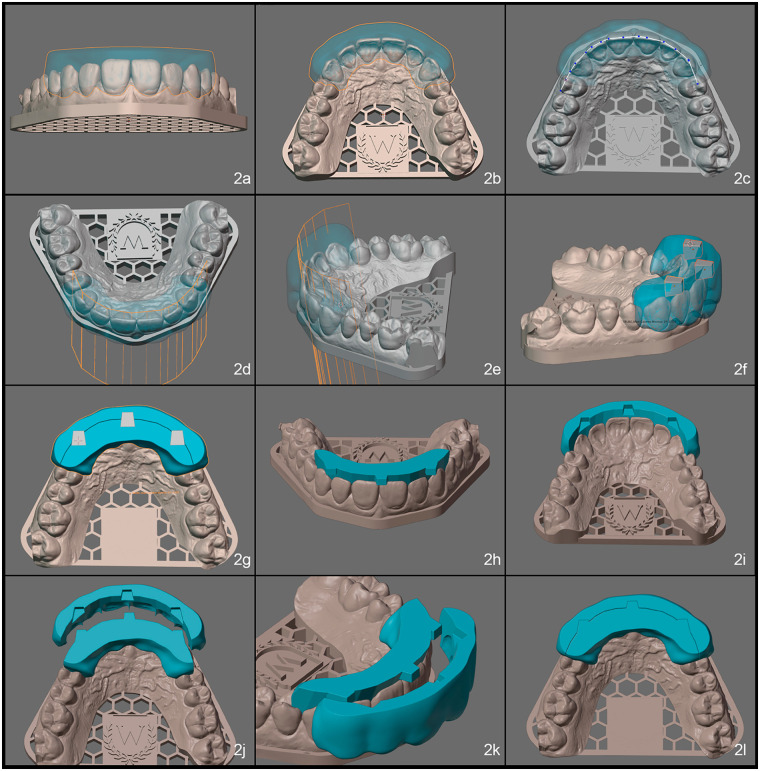
Fabrication workflow of the 3D-printed dual-matrix system in blender software: **(a)** buccal view of the selected extension of the guide. The incisal area was planned to remain flat; **(b)** Incisal view of the guide, showing a convex buccal surface and a concave palatal surface extending to the mesial aspect of the first premolars; **(c)** Selection of the points where segmentation of the buccal and palatal portions would be performed; **(d)** Segmentation path traced through the points selected in Figure 2C; **(e)** Approximately 5° buccal inclination prior to guide segmentation; **(f)** Incorporation of geometric interlocking features within the guide; **(g)** Incisal view showing the segmentation line between the buccal and palatal portions, as well as the overlapping geometric interlocking features assigned to each portion; **(h)** Buccal view of the palatal guide showing the positioning keys projecting buccally beyond the segmentation line of the palatal key; **(i)** Palatal view of the guide showing the receptive slots marked over the vestibular guide in the incisal surface area; **(j)** Incisal view showing the approximation of both keys; **(k)** Lateral view illustrating how the positioning keys of the palatal guide fit into the receptive slots of the vestibular guide; **(l)** Incisal view showing the assembly of both keys and their adaptation until complete seating/closure.

The STL file was then imported into a 3D modeling software (Blender 4.5.1 LTS, Blender Foundation). A full-coverage guiding shell was initially designed over the wax-up, extending from premolar-to-premolar regions and covering the facial, palatal, and incisal aspect (Figures 2a,b). The external surface of this shell was designed to be flat on the incisal aspect, convex on the facial surface, and slightly concave on the palatal surface to facilitate handling and proper seating.

The shell was subsequently segmented into two components at the level of the incisal edge of the digital wax-up, creating a palatal matrix and a vestibular matrix (Figures 2c,d). Importantly, the separation plane was not perpendicular but slightly inclined (approximately 5°) to improve the path of insertion and reduce potential interferences during seating (Figure 2e).

To ensure precise positioning and stability during clinical use, internal blocks were incorporated at the level of the midline during the digital design phase (Figures 2f,g). These structures were subsequently transformed into three positive indexing features (positioning keys) within the palatal matrix (Figure 2h). The corresponding negative counterparts (receptive slots) were incorporated into the vestibular matrix (Figure 2i), enabling precise interlocking with the palatal component and guiding its positioning during assembly (Figures 2j–l). This configuration functions as a controlled positioning system, enhancing reproducibility and ensuring stable, consistent seating of the dual-matrix assembly (Figure 2l).

### 2.3 3D-printing of the dual-matrix system

**Figure 3:**
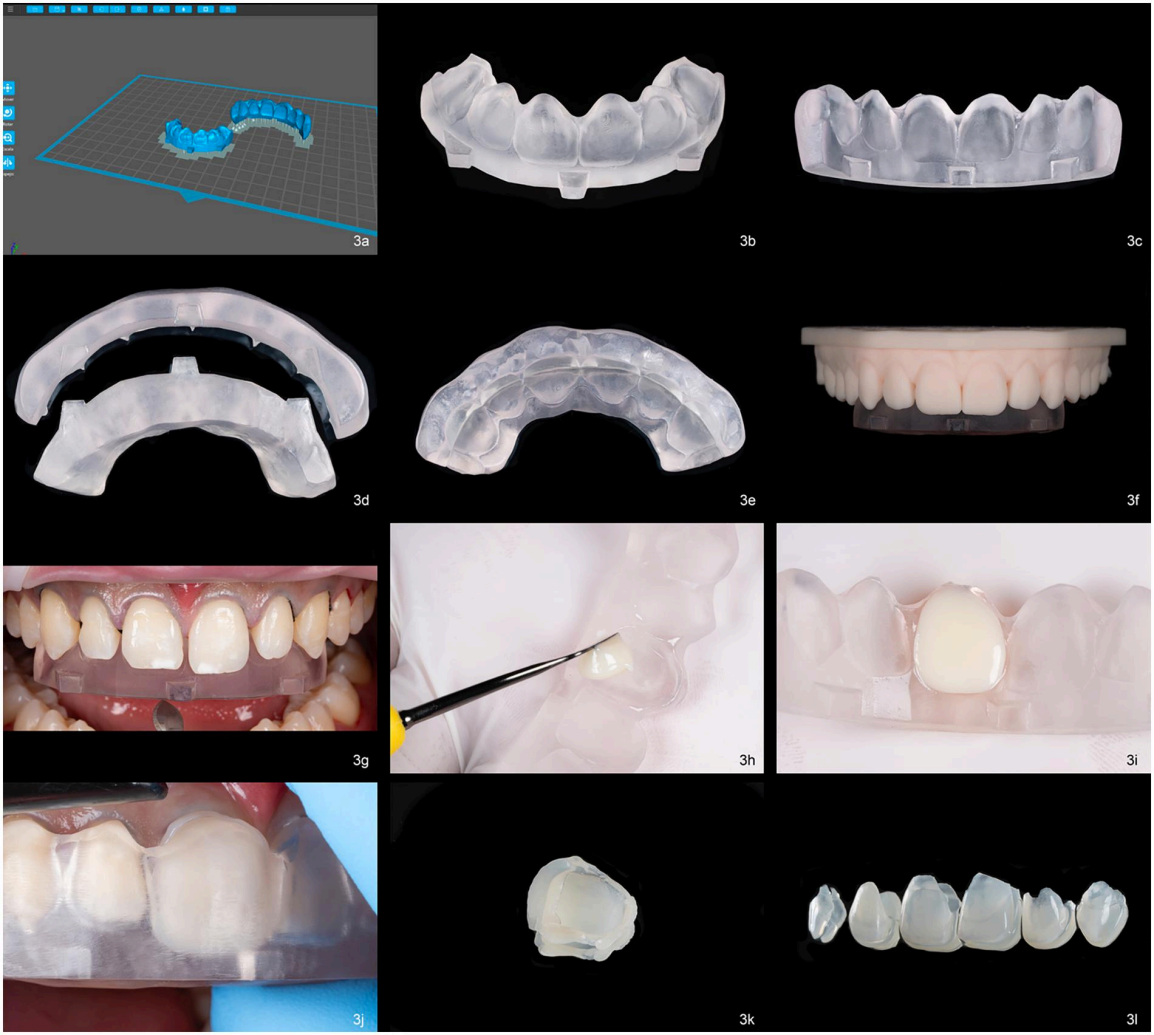
**(a)** Printing workflow of the matrices in Chitubox software; **(b)** Printed palatal key, showing the positioning keys projecting toward the buccal aspect; **(c)** Palatal view of the vestibular key; **(d)** Illustration of both printed keys; **(e)** Assembly of both keys demonstrating precise seating and adaptation between the two matrices; and **(f)** Verification of the fit of the palatal key using the digital wax-up on a 3D-printed model. And clinical application sequence of the use of the 3D-printed dual matrix system for the fabrication of indirect resin composite veneers: **(g)** Placement of the palatal key intraorally in position to verify proper seating and adaptation; **(h)** Application of an enamel-like composite resin layer onto the internal surface of the vestibular key; **(i)** Illustration showing complete coverage of the delimited tooth area by the composite resin within the key; **(j)** Seating of the vestibular key onto the palatal key until complete adaptation and assembly of both matrices was achieved; **(k)** Polymerized composite resin veneer removed from the vestibular key; and **(l)** Image illustrating all completed composite resin veneer restorations.

The dual-matrix system was exported as two independent STL files and processed using a slicing software (Chitubox v1.2.0, CBD-Tech, China) prior to fabrication (Figure 3a). The components were manufactured using a translucent 3D printing resin (BioSplint, Prizma) with a stereolithography (SLA/LCD) 3D printer (Phrozen Sonic Mini 4 K, Phrozen Technology, Taiwan) following to the manufacturer's recommended printing parameters, as summarized in Table 1. BioSplint resin was selected because of its translucency, dimensional stability, and rigidity after post-curing, properties considered advantageous for maintaining matrix geometry and allowing light transmission during the initial polymerization of the composite resin.

**Table 1:** 3D printing parameters used for fabrication of the dual-matrix system.

Parameter	Value
Layer thickness	0.05 mm
Bottom layers	5 layers
Exposure time	5 s
Bottom exposure time	80 s
Transition layers	11
Transition layer exposure decrement	6.25 s
Light-off delay	0 s
Bottom lift distance	6 mm
Lifting distance	6 mm
Retraction distance	6 mm
Bottom lift speed	65 mm/min
Lifting speed	65 mm/min
Bottom retract speed	150 mm/min
Retraction speed	150 mm/min

After printing, both components of the dual-matrix system (palatal and facial matrices) were washed in isopropyl alcohol (IPA, ≥ 97%) using an automated washing unit (Phrozen Wash+, Phrozen Technology, Taiwan) for 5 min under continuous agitation to effectively remove uncured resin residues. Following the cleaning procedure, the matrices were thoroughly dried using compressed air to ensure complete solvent evaporation prior to post-curing.

Subsequently, both matrices were post-cured using a light-curing unit (Phrozen Luna Cure, Phrozen Technology, Taiwan), operating within a wavelength range of 365–405 nm. The components were polymerized for 15 min. Complete polymerization was visually confirmed by the transition of the material from its initial violet color to a transparent appearance (Figures 3b–e).

Additionally, the digital wax-up was 3D printed using a model resin (Model 2.0, Prizma) to obtain a physical model. This step allowed for extraoral verification of the passive seating of the dual-matrix system prior to the clinical procedure, ensuring proper adaptation and stability before intraoral application (Figure 3f).

### 2.4 Fabrication of indirect resin composite veneers

Under relative isolation using a lip retractor (OptraGate, Ivoclar) and a gingival retraction cord placed around the teeth to be restored, the fit and insertion path of both matrices were clinically verified (Figure 3g).

A layer of enamel-shade resin composite (Bleach L Enamel, IPS Empress Direct, Ivoclar) was then applied onto the internal surface of the facial matrix (Figures 3h,i), ensuring complete and homogeneous coverage of the corresponding tooth surfaces. No adhesive procedure was performed at the enamel surface at this stage.

The palatal matrix was first positioned intraorally and stabilized in place. Subsequently, the facial matrix was seated over it, guided by the positive indexing features (positioning keys) and their corresponding negative counterparts (receptive slots), ensuring precise alignment and complete seating of the dual-matrix system without displacement (Figure 3j).

Once both matrices were fully seated, the resin composite was light-cured at 1,200 mw/cm^2^ for 60 s through the transparent facial matrix to ensure adequate polymerization (Bluephase N G4, Ivoclar). After curing, the dual-matrix system was carefully disassembled, and the fabricated indirect resin composite veneer was removed from both the tooth surface and the matrix (Figure 3k).

Extraorally, excess material was meticulously removed using aluminum oxide discs (Sof-Lex XT, Solventum). Subsequently, the restorations were individualized through the application of characterization stains (Violet, Blue, and White, IPS Empress Direct Color, Ivoclar), enhancing incisal opalescence and reproducing the incisal halo effect for each veneer. This characterization procedure was performed individually for each restoration and then all restorations were polished using diamond high-shine polishing rubber wheels (OptraGloss, Ivoclar) (Figure 3l). A Final post-curing was performed before adhesive luting in order to improve mechanical properties.

### 2.5 Adhesive luting procedure

**Figure 4:**
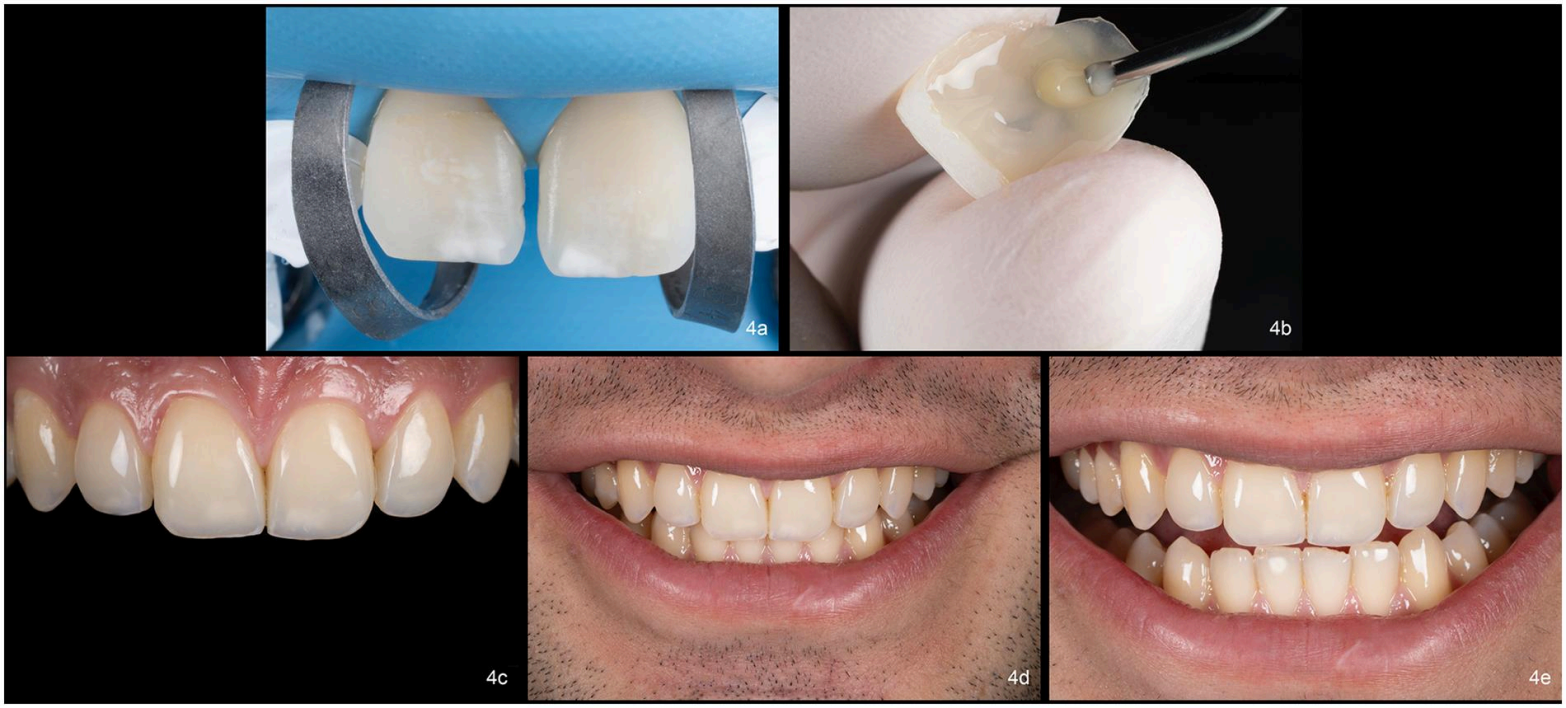
Adhesive luting procedure and final clinical outcome: **(a)** The adhesive luting procedure performed under rubber dam isolation; **(b)** Illustrative image showing the application of the resin cement after surface treatment of the indirect composite veneer restoration; **(c)** Intraoral view of the final maxillary outcome; **(d)** Dento-labial view on maximum intercuspation (MIP) of the final result; and **(e)** Final smile result image.

Under rubber dam isolation (Figure 4a), each tooth was etched with 37% phosphoric acid (Total Etch, Ivoclar) for 30 s, thoroughly rinsed with water, and gently air-dried. A thin layer of a universal adhesive (Tetric N Bond Universal, Ivoclar) was applied, followed by air thinning for 20 s to evaporate solvents, and subsequently light-cured for 20 s.

The internal surface of each resin composite veneer was initially airborne-particle abraded using 27 *μ*m aluminum oxide particles (Aquacare, Valopex, UK) for 10 s at a distance of 1 cm. The restorations were then cleaned in distilled water using an ultrasonic bath for 5 min, followed by the application of a silane coupling agent (Monobond N, Ivoclar). Each veneer was then loaded with a light-cured resin cement (Variolink Esthetic LC, Light shade, Ivoclar) (Figure 4b).

The restoration was positioned onto the corresponding tooth using gentle finger pressure, and excess cement was removed using a fine brush. Polymerization was performed for 60 s at an irradiance of 1,200 mw/cm^2^. After curing, any remaining excess cement was carefully removed using a #11 scalpel blade, and the margins were polished using diamond polishing rubbers (OptraGloss, Ivoclar)

Each restoration was cemented individually. Final images are presented in Figures 4c–e.

## 3 Discussion

The fabrication of indirect resin composite veneers remains a technique-sensitive and operator-dependent procedure, particularly when morphology is established through freehand layering (2). In such approaches, the accurate transfer of the diagnostic wax-up is often compromised, resulting in limited reproducibility and variability in clinical outcomes. Although matrix-assisted techniques have been introduced to improve morphological control, conventional systems still present limitations in precision and handling (16, 17). Therefore, more standardized and controlled approaches are required to enhance the predictability of the final restoration.

To address these limitations, the present case introduces a novel strategy based on a fully digital workflow integrating CAD design and a 3D-printed dual-matrix system with interlocking features. To the best of our knowledge, no previous study has described a fully digitally designed and 3D-printed dual-matrix system with an interlocking mechanism for the guided fabrication of indirect composite veneers. This design enables the precise transfer of the diagnostic wax-up morphology from the digital environment to the clinical setting, facilitating guided positioning during insertion. Additionally, the matrices can be fully defined during the digital planning stage, allowing preoperative control of their thickness, geometry, and insertion path. Collectively, these features reduce operator dependency and improve consistency and reproducibility of the restorative procedure.

Recent reports have demonstrated the successful application of digitally guided injection techniques using transparent matrices for direct composite restorations, highlighting their ability to accurately transfer the diagnostic wax-up improve morphological reproducibility during treatment (14, 15). These approaches represent an important advancement in minimally invasive esthetic dentistry by reducing operator dependency and facilitating predictable restoration design. However, they are primarily intended for direct restorations, in which the restorative material is polymerized and finished intraorally. In contrast, the present technique extends the concept of digitally guided morphology transfer for the fabrication of indirect composite veneers. This workflow allows extraoral characterization, finishing and polishing, and post-curing procedures, which may provide advantages in terms of surface quality, degree of conversion, and long-term mechanical performance. Nevertheless, further laboratory and clinical studies are required to validate these potential benefits.

From an adhesive perspective, this level of control over restoration positioning may contribute to a more uniform resin cement layer and improved adaptation at the tooth-restoration interface. These factors are critical in adhesive dentistry, as variations in cement thickness and seating discrepancies can influence polymerization shrinkage stress, interfacial integrity, and ultimately the longevity of bonded restorations (10, 11). In addition, airborne-particle abrasion was restricted to the intaglio surface to enhance micromechanical retention prior to adhesive cementation. Because this treatment affects only a superficial layer of the restoration, it is not expected to compromise the bulk mechanical properties of the composite veneer.

Conventional translucent silicone matrices, while widely used, are inherently flexible and susceptible to deformation during intraoral placement (18). The seating pressure required to stabilize these matrices may induce dimensional changes, potentially compromising the accurate replication of the wax-up morphology (19). In contrast, the proposed 3D-printed matrices exhibit greater rigidity and structural stability, allowing a more consistent transfer of surface details (17). Furthermore, silicone matrices often require intraoral trimming to achieve proper adaptation, increasing procedural variability and reliance on operator skill (16). From an optical perspective, silicone matrices generally require a minimum thickness to maintain stability, which may reduce light transmission and affect polymerization efficiency (20). Conversely, 3D-printed translucent matrices can be designed with controlled and reduced thickness, optimizing light transmission while preserving structural integrity without deformation, while maintaining sufficient rigidity and dimensional stability throughout seating and polymerization procedures.

Additionally, improved light transmission through thinner and more homogeneous matrices may enhance the initial polymerization of the composite during the intraoral phase, ensuring adequate stabilization prior to removal (20). As the restorations are subsequently post-cured extraorally to increase the degree of conversion, intraoral light exposure primarily serves to achieve sufficient pre-curing without compromising morphology or handling (7). Moreover, most composite polymerization shrinkage occurs during the fabrication stage of the restoration. Consequently, the definitive adhesive interface is established only after post-curing and adhesive cementation, potentially reducing the influence of composite shrinkage on the final tooth-restoration interface (10, 11).

From a clinical standpoint, the dual-matrix system facilitates improved control over tooth morphology, contours, and proximal contacts by enabling a guided and reproducible transfer of the planned design. This is particularly advantageous in high-demand esthetic situations such as diastema closure (17), where symmetry and proportional accuracy are critical. Additionally, the indirect approach allows for enhanced surface finishing and improved mechanical properties through extraoral post-polymerization (7). Although IPS Empress Direct is primarily indicated for direct restorations, additional extraoral post-curing may increase the degree of conversion and further support its application in indirect restorative procedures (2, 8, 9). The technique may also reduce chairside time and decrease reliance on advanced manual layering skills. Although its implementation requires familiarity with digital workflows and access to CAD software and 3D-printing technology, it represents a valuable tool for both experienced clinicians and those with limited expertise in freehand composite techniques.

This case report presents inherent limitations, including its single-case design and the absence of quantitative evaluation of accuracy, marginal adaptation, and reproducibility. Furthermore, no *in vitro* or clinical studies have yet validated the performance of this specific technique. Its application is also dependent on access to digital equipment and calibrated 3D-printed systems. Future studies are required to assess its precision, mechanical performance, and long-term clinical outcomes. Future research should also investigate the influence of this guided system on adhesive parameters such as cement thickness distribution, bond strength, polymerization stress, and marginal integrity, in order to validate its potential benefits in adhesive restorations.

Despite these limitations, the proposed technique represents a promising and predictable alternative for the fabrication of indirect resin composite veneers within a modern digital workflow. The patient reported satisfaction with both the treatment process and the final esthetic outcome.

## 4 Conclusion

The 3D-printed dual-matrix system described in this case report enables a controlled and reproducible transfer of the diagnostic wax-up for the fabrication of indirect resin composite veneers. By integrating a fully digital workflow with guided clinical execution, this approach may enhance the predictability and consistency of esthetic outcomes, and may also contribute to improved control of restoration seating and the adhesive interface. Further studies are required to validate its clinical performance and generalizability.

## Data Availability

The original contributions presented in the study are included in the article/Supplementary Material, further inquiries can be directed to the corresponding author/s.
